# Pore-Level Multiphase Simulations of Realistic Distillation Membranes for Water Desalination

**DOI:** 10.3390/membranes12111112

**Published:** 2022-11-08

**Authors:** Tobias Jäger, Athanasios Mokos, Nikolaos I. Prasianakis, Stephan Leyer

**Affiliations:** 1 Department of Engineering, Faculty of Science, Technology and Medicine, University of Luxembourg, L-1359 Luxembourg, Luxembourg; 2 Transport Mechanisms Group, Laboratory for Waste Management, Paul Scherrer Institute, 5232 Villigen PSI, Switzerland

**Keywords:** membrane distillation, water treatment, lattice Boltzmann method, multi-phase flow

## Abstract

Membrane distillation (MD) is a thermally driven separation process that is operated below boiling point. Since the performance of MD modules is still comparatively low, current research aims to improve the understanding of the membrane structure and its underlying mechanisms at the pore level. Based on existing realistic 3D membrane geometries (up to 0.5 billion voxels with 39nm resolution) obtained from ptychographic X-ray computed tomography, the D3Q27 lattice Boltzmann (LB) method was used to investigate the interaction of the liquid and gaseous phase with the porous membrane material. In particular, the Shan and Chen multi-phase model was used to simulate multi-phase flow at the pore level. We investigated the liquid entry pressure of different membrane samples and analysed the influence of different micropillar structures on the Wenzel and Cassie–Baxter state of water droplets on rough hydrophobic surfaces. Moreover, we calculated the liquid entry pressure required for entering the membrane pores and extracted realistic water contact surfaces for different membrane samples. The influence of the micropillars and flow on the water-membrane contact surface was investigated. Finally, we determined the air–water interface within a partially saturated membrane, finding that the droplet size and distribution correlated with the porosity of the membrane.

## 1. Introduction

Freshwater scarcity is already a severe problem for many countries in the world and will deteriorate in the future due to the climate crisis [1]. However, many arid areas have both access to seawater and high solar irradiance. Therefore seawater desalination is a promising approach for these countries. The most used seawater desalination technologies rely on thermal distillation and reverse osmosis. Common thermal separation processes include multi-effect distillation, a low-pressure steam process, and multi-stage flash evaporators [2]. Nevertheless, these technologies require a lot of energy and a sufficient power supply infrastructure [3].

Membrane distillation (MD) is a thermally driven separation process that is operated below the boiling point of water and is capable of providing significant contributions to the problem of water scarcity in the world [4]. In MD, evaporation is used to extract pure water from saline or brackish water. Compared to the aforementioned technologies, MD has the advantage of small investment and low operating costs as it can be driven by non-concentrated solar energy [5] or waste heat [4]. However, the overall efficiency and output production rate of MD modules are still too small to be competitive [6,7].

**Figure 1:**
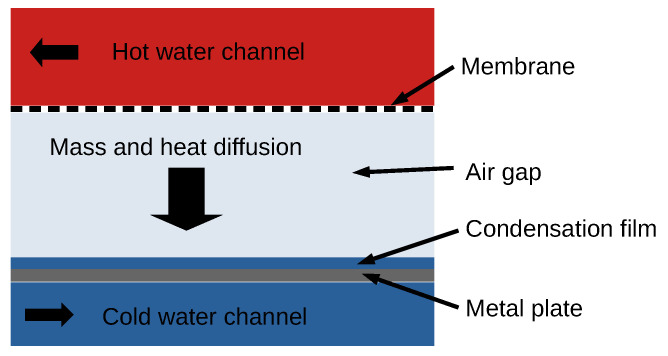
Sketch of an AGMD module.

The key to improving the design, performance, and efficiency of MD modules is to better understand the underlying mechanisms that govern the separation processes. Several different MD designs have been developed. A very promising design for seawater distillation is the air gap membrane distillation (AGMD) module. In this approach, the hot feed solution is in direct contact with a hydrophobic membrane, but in contrast to direct contact membrane distillation, an air layer separates the membrane and the condenser (See Figure 1). The water vapour diffuses through the membrane and the air gap to condense on a cold surface. The hydrophobicity of the membrane prevents the saltwater from ingressing within the membrane. Salt contaminants cannot be transported via the vapour phase and only water molecules in vapour form can cross the membrane [8].

The main benefit of AGMD is the thermal insulation properties of the air gap, which reduce heat loss. On the downside, the air gap represents an additional mass transport pathway, which has an effect on the membrane throughput [4]. The salt concentration at the evaporation interface is also increased due to water evaporation, which can lead to the precipitation of salt and other pollutants and the blockage of the membrane pores. Specific membrane structures such as micropillars and increased hydrophobicities can reduce scaling and prolong the life cycle of a membrane [9,10].

The phase change and mass transport mechanisms take place within the membrane’s microporous structure. An important dimensionless number for characterising the flow regime is the Knudsen number ($\mathrm{Kn}=\frac{\lambda }{L}$), where λ is the mean free path length of the particles and *L* is the characteristic length scale of the flow [11]. The Knudsen number can be used to define four flow regimes: the continuum (Kn<0.01), slip flow (0.01<Kn<0.1), transitional flow (0.1<Kn<10), and free molecular flow (Kn>10). For MD applications, *L* represents the pore diameter of the membrane (200–450 nm), and with respect to the vapour phase, this results in a Knudsen number of Kn≈1 [12].

Since assessing the flow on the microscopic level through experimental methods is complex, little is known about the interaction of liquid, gas, and hydrophobic membrane material at the pore level. To increase the efficiency of MD modules a better understanding of the underlying mechanisms, such as evaporation at the pore level, would be beneficial. For this reason, detailed numerical modeling of the mechanisms occurring in MD is required.

Evaporation as a non-equilibrium process is a complex phenomenon with temperature jumps observed at the liquid–gas interface where evaporation occurs. Different theories, such as the Hertz–Knudsen equation and statistical rat theory (SRT), were developed to calculate the evaporation flux [13,14].

The size of the evaporation flux depends among other parameters on the vapour pressure above the liquid–gas interface, the surface area of the liquid–gas interface, and the salt content in the water. The larger surface areas of the liquid–vapour interface will lead to higher evaporation fluxes. The basis for the evaporation models mentioned above is a static liquid–gas interface, which needs to be determined before the model is applied.

The fluid dynamics of this case can be expressed either through a continuum-based approach or a particle-based approach. For the continuum approach, macroscopic quantities such as density and velocity are used to describe the flow, whereas the particle approach takes into account the individual particles (atoms, molecules) and their interactions. For domains, orders of magnitude larger than the mean free path of the particle continuum-based approaches, such as the Navier–Stokes equation and classical computational fluid dynamics (CFD) solvers, provide a viable description of the flow. For 3D micro- and nano-scale complex microstructures, as is the case for the considered membranes, the lattice Boltzmann (LB) framework is a promising approach [15,16].

The LB method describes the flow on a mesoscopic level up to the continuum level and allows for the simulation of the flow in the slip flow regime (Kn<0.1). The LB method is based on kinetic gas theory and the time evolution of the one-body distribution function,$f(\vec{x},\vec{v},t)$ which is a probability density function. $f\left(\vec{x},\vec{v},t\right)\;d\vec{x}\;d\vec{v}$ is the mean number of particles in a phase-space volume $d\vec{x}\;d\vec{v}$ at position $\vec{x}$ with velocity $\vec{v}$ at time *t*. For the LB method, the phase space is discretized, ending up in a domain with discrete cubic cells and discrete velocities.

When membrane distillation modules are modeled numerically, the membrane and the transport of the water vapour through the membrane are typically modeled using the dusty gas model and a flat liquid–membrane interface. For the dusty gas model, only membrane properties, such as porosity and tortuosity, as well as the viscosity of the gas mixture, are required as input for the calculation of the macroscopic flux through the membrane. A microscopic description of the membrane geometry at the pore level is not taken into account within the scope of the dusty gas model [8,17].

To obtain a detailed picture of the liquid–membrane interface, we use high-resolution membrane geometries, which are resolved at the pore level [12], and a multi-phase LB method. In order to validate the model, we simulate typical benchmarks such as the contact angle and Laplace law benchmark. To ensure the correct interaction with the rough membrane material, we validate the influence of micropillar structures on the apparent contact angle in both the Cassie–Baxter and the Wenzel states [18,19] and identify the liquid entry pressure (LEP) needed for the water to move into the pores of the hydrophobic membrane for both a cylindrical and realistic geometry. The LEPs for the realistic membrane samples are compared to the values from the manufacturers. Finally, we use the validated model to determine realistic water–membrane contact surfaces and investigate the droplet distribution within 3D membrane geometries.

## 2. Materials and Methods

### 2.1. Membrane Geometry

Experimentally determined [12] membrane geometries were used. A summery of the membrane samples can be seen in Table 1. Cramer et al. [12] used ptychographic X-ray-computed tomography to map the 3D membrane structure of four commercially used membranes at an unprecedented resolution. Pictures of the full membrane geometry are available in [12]. The experiments were conducted at the cSAXS beamline of the Swiss Light Source, PSI, Switzerland. The membrane material was PTFE and the manufacturer values of the porosity were 85% for samples 1, 3, and 4 and 80% for sample 2. The X-ray device used photons with an energy of 6.2keV achieving a spatial cubic voxel size of $\Delta^{SI}x=0.03899\;\mu m$ and a total voxel number of about 0.5 billion voxels per sample. The electron density difference between the membrane material and epoxy (pore space in the membrane) can be used to create a binary (membrane and void) 3D image of the membrane by applying a threshold. For samples 2, 3, and 4, the porosity value based on the ptychographic X-ray-computed tomography matched the manufacturer values [12]. Moreover, Cramer et al. [12] calculated membrane characteristics such as tortuosity and permeability based on single-phase LB simulations with a pressure gradient. Note that samples 1 and 4 originated from different parts of the same membrane.

### 2.2. Numerical Method

**Figure 2:**
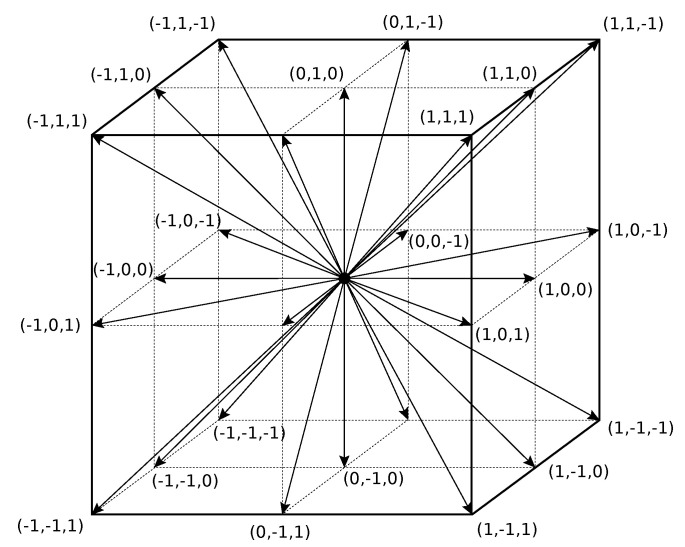
D3Q27 lattice with 27 discrete velocities ${\vec{c}}_{i}$.

In this paper, the D3Q27 LB method (see Figure 2) was applied with 27 discrete velocities (${\vec{c}}_{i}$, i=0..26) in a 3D space [20].

The evolution of the discrete density distribution function can be written as:

(1) $$f_{i}\left(\vec{x}+{\vec{c}}_{i}\Delta t,t+\Delta t\right)-f_{i}\left(\vec{x},t\right)=-\frac{1}{\tau }\left[f_{i}\left(\vec{x},t\right)-f_{i}^{eq}\left(\rho ,\vec{v},\vec{x},t\right)\right]$$

The term on the right is the Bhatnagar–Gross–Krook approximation of the collision operator, reflecting the inter-particle collisions in the fluid [21]. The relaxation time τ is linked to the viscosity of the fluid. In the LB method, the discrete one-body distribution function $f_{i}$ is relaxed towards a local equilibrium $f_{i}^{eq}$. The term on the left represents the propagation of the density distribution function. In this step, the $f_{i}$s are streamed to their respective neighbouring cells.

The LB method is usually not simulated with SI units but as a similarity problem. For our LB simulation, we used the common LB unit convention: the time step (ts) was set to one, the lattice spacing (Δx) was set to one lattice unit (lu), and the mass of one fluid particle (*m*) was set to one mass unit (mu). This way, the LB unit convention gave Δt=1ts, Δx=1lu, m=1mu. Therefore, the speed of sound equalled $c_{s}=\frac{1}{\sqrt{3}}\frac{\mathrm{lu}}{\mathrm{ts}}$ for the D3Q27 lattice. As is typically done for such multi-phase LB simulations, e.g., [22], for increased stability and faster numerical convergence we chose τ=1ts for our simulations. This corresponds to a kinematic viscosity of $1/6\;{\mathrm{lu}}^{2}/\mathrm{ts}$ ($\nu =c_{s}^{2}\left(\tau -0.5\right)\Delta t$). In SI units, this corresponds to $\Delta t^{SI}=0.087\;\mathrm{ms}$ and $\Delta^{SI}x=0.03899\;\mu m$ for the membrane geometries mentioned above.

In order to initialize the LB simulation so that it corresponded to the real physical problem, the relevant governing dimensionless numbers must be equal. The problems studied here are governed by the Laplace and Bond dimensionless numbers. The Laplace number is typical for free-surface fluid dynamic flows (multi-phase flow). It relates the momentum and surface tension forces to viscous forces ($\mathrm{La}=\frac{\gamma \;\rho_{l}\;L}{{\left(\eta_{l}\right)}^{2}}$) [23] and is used to map the surface tension LB units to the SI units. γ represents the surface tension, $\rho_{l}$ is the density of the liquid, $\eta_{l}$ is the dynamic viscosity of the liquid and *L* is the characteristic length.

(2) $$\gamma^{SI}=\gamma \frac{\rho_{l}\;{\left(\eta_{l}^{SI}\right)}^{2}}{\Delta x^{SI}\;\rho_{l}^{SI}\;{\left(\eta_{l}\right)}^{2}}$$

The Bond number ($\mathrm{Bo}=\frac{\Delta \rho gL^{2}}{\gamma }$) describes the ratio of gravitational to capillary forces [23]. For the membranes assessed, the Bond number is Bo≈0.0001, which means that gravitation is negligible. To convert the time step to SI units, the capillary number can be used $\Delta t^{SI}=\frac{\mu \Delta x^{SI}\gamma }{\gamma^{SI}\mu_{SI}}$. This results in $\Delta t^{SI}=0.23\cdot 10^{-9}\;s$ for the assessed membranes. To convert the pressure from LB units to SI units, the following expression was used: $p^{SI}=p\cdot \frac{\gamma^{SI}}{\gamma \cdot \Delta x^{SI}}$.

The LB method is designed to reproduce the Navier–Stokes equation in the hydrodynamic limit [20]. Macroscopic quantities such as density and velocity can be calculated by summing up the discrete populations $f_{i}$ and discrete velocities $c_{i}$ [20].

(3) $$\rho =\sum_{i=0}^{26}f_{i}$$

(4) $$\vec{v}=\frac{1}{\rho }\sum_{i=0}^{26}f_{i}{\vec{c}}_{i}$$

In this case, the guided equilibrium (GE) model proposed in [20,24] was used. The GE function was found by minimizing the entropy function under four constraints. These constraints ensured the conservation of mass, momentum, and energy, as well as imposed an additional constraint on the pressure tensor. This fourth constraint was a “necessary (but not sufficient) condition in order to recover the Navier-Stokes equations” [20]. The results can be written in the following form:

(5) $$f_{i}^{eq}\left(\rho ,\vec{v}\right)=\rho \prod_{\alpha =x,y,z}\frac{1-2c_{i\alpha }^{2}}{2^{c_{\alpha }^{2}}}\left(c_{i\alpha }^{2}-1+c_{i\alpha }v_{\alpha }+v_{\alpha }^{2}+T\right)$$

The Shan–Chen multi-phase model was chosen [22,25,26]. The interaction between fluid particles was achieved by including the following force:

(6) $$\vec{F}=-G\psi \left(\vec{x},t\right)\sum_{i=0}^{26}w_{i}\psi \left(\vec{x}+{\vec{c}}_{i}\Delta t,t\right){\vec{c}}_{i}$$

This leads to the non-ideal equation of state shown in Equation (7). The function ψ(ρ) describes the interaction potential and depends on the density, whereas *G* describes the interaction strength between the fluid particles and allows the surface tension to be adjusted. According to [26,27], the following form of ψ was chosen: $\psi \left(\rho \right)=\psi_{0}\exp\left(-\frac{\rho_{0}}{\rho }\right)$, $\psi_{0}=4$, $\rho_{0}=200$, and RT=1/3. This results in a phase separation below the critical value $G_{crit}=-92.4$. In this work, two different values were used: (a) G=−120.0, which yields a liquid density of $\rho_{l}=524.4\;\mathrm{mu}\;{\mathrm{lu}}^{-3}$ and a gas density of $\rho_{g}=85.7\;\mathrm{mu}\;{\mathrm{lu}}^{-3}$ in LB units for a flat liquid–gas interface [27], and (b) G=−180.0, leading to a liquid density of $\rho_{l}=1027.81\;\mathrm{mu}\;{\mathrm{lu}}^{-3}$ and a gas density of $\rho_{g}=52.82\;\mathrm{mu}\;{\mathrm{lu}}^{-3}$ in LB units for a flat liquid–gas interface.G=−120.0 is a typical value found in the literature andfor G=−180.0, the above-mentioned membrane geometries result in the desired surface tension of $\gamma^{SI}=0.073\;N/m$ (see Table 2 and Equation (2)). This corresponds to the surface tension of pure water at $16.5{\;}^{\circ }C$.

(7) $$p=\rho RT+\frac{GRT}{2}{\left(\psi \left(\rho \right)\right)}^{2}$$

We note that for the results presented in this paper, which are relevant to the shape of the liquid phase within the porous medium, the capillary forces are dominant rather than the density ratio between the liquid and gas phases. For simulations where the liquid phase’s dynamic motion and interaction with the gas phase are of central importance, the model can be adjusted accordingly to capture the relevant physics. For the interaction between the fluid and solid, the following force is introduced, with the function $s(\vec{x})$ giving 1 for solid nodes and 0 for fluid nodes [22,27].

(8) $${\vec{F}}_{ads}=-G_{ads}\psi \left(\vec{x},t\right)\sum_{i=0}^{26}w_{i}s\left(\vec{x}+{\vec{c}}_{i}\Delta t,t\right){\vec{c}}_{i}$$

The parameter $G_{ads}$ correlates linearly with the contact angle for a given *G* value, as shown in the contact angle benchmark in Section 3.2. $G_{ads}$ allows tuning the contact angle of a liquid droplet on the flat solid phase.

Additionally, a non-slip (bounce-back) boundary condition was employed for the fluid–solid interaction. The membrane samples in Table 1 only represent a small cutout of a membrane sheet typically used in MD. To mimic a much larger membrane sheet, periodic boundary conditions (PBC) were employed at the border of the domain. If not stated otherwise, PBC were used in all directions, *x*, *y*, and *z*, for our LB simulations. We also implemented density (pressure) boundary conditions for the LEP. Similar to [22], we used Zou and He [28] boundary conditions adapted for the D3Q27 lattice [29]. The total force is given by:

(9) $${\vec{F}}_{tot}=\vec{F}+{\vec{F}}_{ads}+{\vec{F}}_{exe}$$

${\vec{F}}_{exe}$ represents an external force such as gravity or a pressure gradient. To incorporate the force in the LB method, Shan and Chen [25,26] modified Equation (4):

(10) $$\vec{v}=\frac{1}{\rho }\sum_{i=0}^{26}f_{i}{\vec{c}}_{i}+\frac{\tau {\vec{F}}_{tot}}{\rho }$$

If not stated otherwise, the convergence criterion in Equation (11) was $1\cdot 10^{-6}$ or better for the simulation.

(11) $${\mathrm{mean}}_{x,y,z}\left(\frac{|\rho^{i}\left(x,y,z\right)-\rho^{i-1}\left(x,y,z\right)|}{\rho^{i-1}\left(x,y,z\right)}\right)$$

For the simulations, a high-performance computing code was adapted [30]. The code uses a hybrid CUDA-MPI programming layout, which enables it to be executed on several Nvidia GPUs in parallel. In the D3Q27 LB method, the evolution of a 27-distribution function has to be calculated at each lattice node. This includes three major steps: collision, streaming, and force calculation. Since the method is explicit and all calculations are local, the multithread parallelization within the GPU can be employed efficiently. For the geometries examined, several GPUs in parallel were used, as the required amount of device memory scaled linearly with the total number of voxels in the domain. During the streaming step and the force calculation, data were exchanged between neighbouring lattice nodes and thus between different computing nodes; therefore, the data transfer between different GPUs was realized with OpenMPI.

## 3. Results and Discussion

### 3.1. Surface Tension—Young–Laplace Benchmark

**Figure 3:**
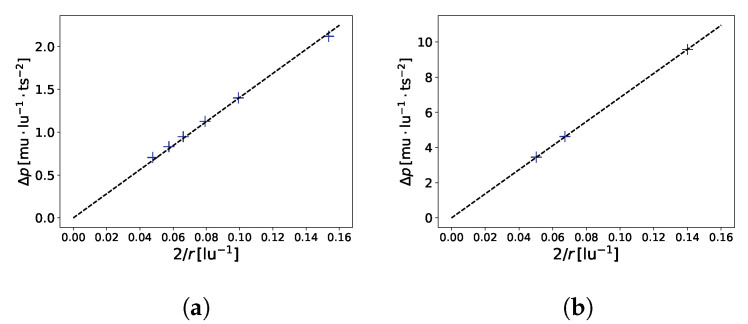
Laplace law benchmark ($\Delta p=\gamma \cdot \frac{2}{r}$). In (**a**), G=−120.0, $R^{2}=0.9972$, $\gamma =14.0409\;\mathrm{mu}\cdot {\mathrm{ts}}^{-2}$, where $R^{2}$ is the coefficient of determination. In (**b**), G=−180.0, $R^{2}=0.999960$, $\gamma =68.45\;\mathrm{mu}\cdot {\mathrm{ts}}^{-2}$. Simulations were performed on a 100 × 100 × 100 domain.

A typical benchmark for a multi-phase model is the verification of the Young–Laplace equation ($\Delta p=\gamma \cdot \frac{2}{r}$), which relates the radius *r* of a bubble to the pressure difference Δp between the liquid and gaseous phases. Figure 3 shows the results of the benchmark, which are in good agreement with the theory. This benchmark also allows for the determination of the surface tension of the gas–liquid interface. For the aforementioned model, we found $\gamma =14.0409\;\mathrm{mu}\cdot {\mathrm{ts}}^{-2}$ (in LB units) for G=−120.0 and $\gamma =68.45\;\mathrm{mu}\cdot {\mathrm{ts}}^{-2}$ (in LB units) for G=−180.0.

### 3.2. Wettability—Contact Angle Benchmark

**Figure 4:**
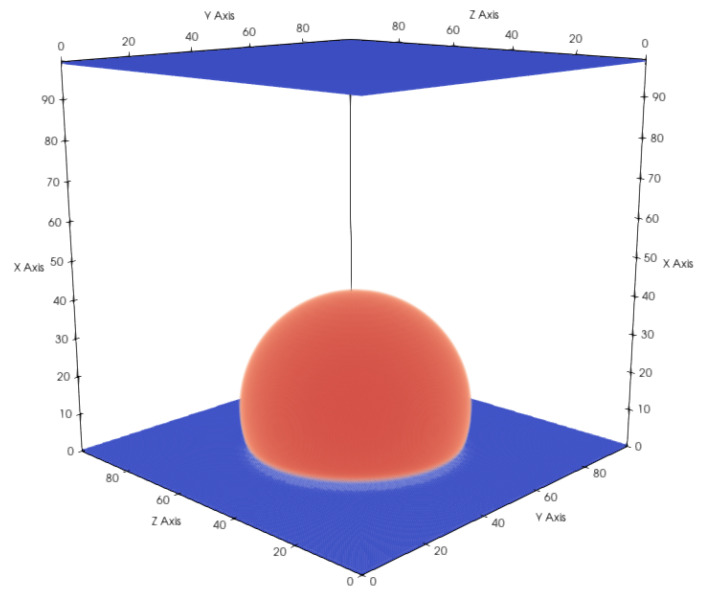
Droplet on a flat hydrophobic surface with a total domain size of 100×100×100 voxels. Gaseous phase is not shown for better visibility. Contact angle changes depending on the hydrophobicity, which can be controlled with $G_{ads}$ and *G*. $G_{ads}=-157.16$ and G=−120.0 result in a contact angle of $110.6^{\circ }$.

An important property for a multi-phase flow in contact with a solid is the wettability of the surface, which is directly related to the contact angle α of a droplet on a flat surface. The contact angle describes the degree of the hydrophobic/hydrophilic properties of the surface and is dependent not only on the chemical composition of the solid material but also on the solid surface characteristics. The contact angle can be set by adjusting the adsorption coefficient $G_{ads}$. In the simulations, we initially placed a cubic liquid droplet on a flat surface and let it equilibrate until the simulation reached a static state (see Figure 4).

**Figure 5:**
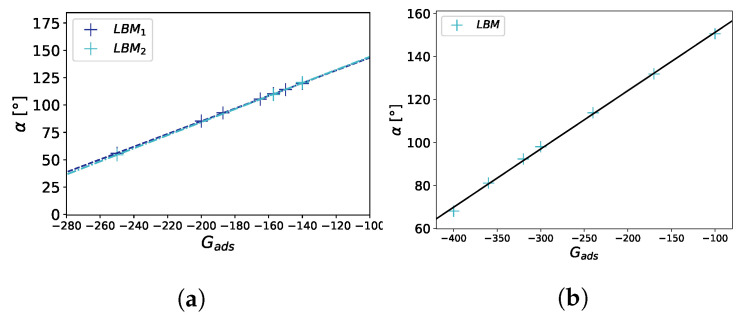
Contact angle benchmark on a flat surface. The contact angle α is plotted against the strength of the fluid–solid interaction $G_{ads}$. In (**a**), G=−120.0, $LBM_{1}$, and $LBM_{2}$ correspond to the different droplet sizes in the LB simulation ($V_{2}\approx 15625\;{\mathrm{lu}}^{3}$, $V_{1}\approx 91125\;{\mathrm{lu}}^{3}$). For a linear fit $\alpha =m\cdot G_{ads}+b$, we obtain for the two droplet sizes $R_{1}^{2}=0.999875$, $m_{1}=0.5808$, $b_{1}=201.36$, $R_{2}^{2}=0.999882$, $m_{2}=0.5973$, $b_{2}=203.78$, where $R_{i}^{2}$ is the coefficient of determination. In (**b**), G=−180.0. Simulations were performed on a 100 × 100 × 100 domain.

To determine the contact angle from the calculation results, we first extracted the points in the vicinity of the liquid–gas interface using the condition $\frac{\rho \left(x,y,z\right)-\rho_{c}}{\rho_{c}}<0.01$ and fitted a sphere to these points. Finally, the expression $\cos\alpha =-\frac{x_{c}-b}{r}$ was used to calculate the contact angle, with $x_{c}$ being the center of the sphere, *r* the radius of the sphere, and *b* the x position of the surface. The numerically measured linear dependency of α on $G_{ads}$ is in agreement with the results of Peng et al. [22]. We also observed that the contact angle was independent of the droplet size (see Figure 5).

### 3.3. Cassie–Baxter and Wenzel States

It is well known that for a droplet on a surface, the (apparent) contact angle also depends on the surface structure. One can distinguish between Cassie–Baxter (CB) and Wenzel states [19,31]. For the CB state, gas is trapped in the gaps created by the surface roughness, with the result that the liquid droplet remains at a higher level and does not penetrate the rough surface. For the Wenzel state, no gas is trapped at all and the liquid is in direct contact with the rough solid phase. It is therefore important to identify the state of the membrane in order to determine the correct contact angle. This step also serves as an additional benchmark to ensure the correct behavior on rough surfaces such as the membrane material studied later in this work.

Cassie and Baxter [19] developed an expression (Equation (12)) that relates the contact angle on a flat surface (α) to the apparent contact angle ($\alpha_{app}$) on a rough surface of the same material for a droplet in the CB state. The parameter $f_{SL}$ determines the fraction of the bottom surface of the liquid droplet in contact with the solid. Based on the micropillar structure, one can calculate $f_{SL}=\frac{A_{top}}{P^{2}}$, where $A_{top}$ is the flat top surface area of a pillar and *P* is the pitch between pillars (periodicity).

(12) $$\cos\alpha_{app}^{theor}=f_{SL}\left(\cos\alpha +1\right)-1$$

Wenzel [31] developed a model describing a droplet in the Wenzel state. The apparent contact angle is computed through

(13) $$\cos\alpha_{app}^{theor}=R_{f}\cos\alpha$$

where $R_{f}=1+\frac{A_{l}}{P^{2}}$ relates the total surface area of the roughness to its projection on a flat plane and $A_{l}$ is the lateral surface of the pillar. Contact angles in the CB state are measured with respect to the plane above the roughness, whereas contact angles in the Wenzel state are measured with respect to the plane on the bottom of the roughness.

**Figure 6:**
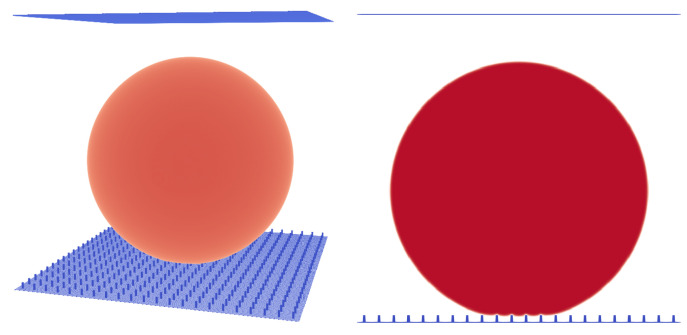
Micro pillar structure with P=17lu, pillar height H=8lu, and $A_{top}=3\times 3\;{\mathrm{lu}}^{2}$. A droplet in the Cassie–Baxter state. Liquid phase is shown in red and micropillar structure is shown in blue.

**Figure 7:**
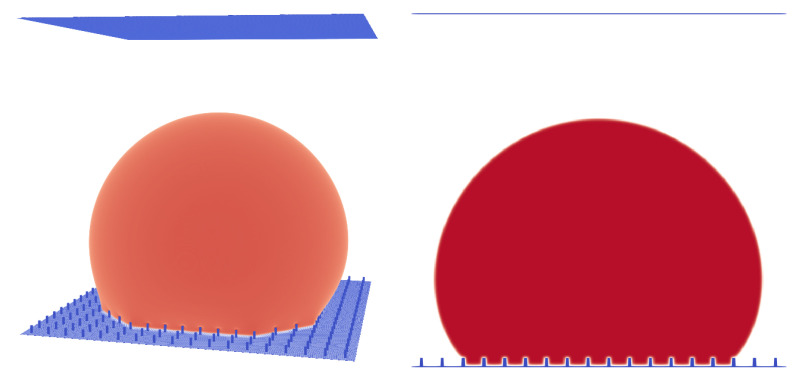
Micro pillar structure with P=20lu, pillar height H=8lu, and $A_{top}=3\times 3\;{\mathrm{lu}}^{2}$. A droplet in the Wenzel state. Liquid phase is shown in red and micropillar structure is shown in blue.

**Figure 8:**
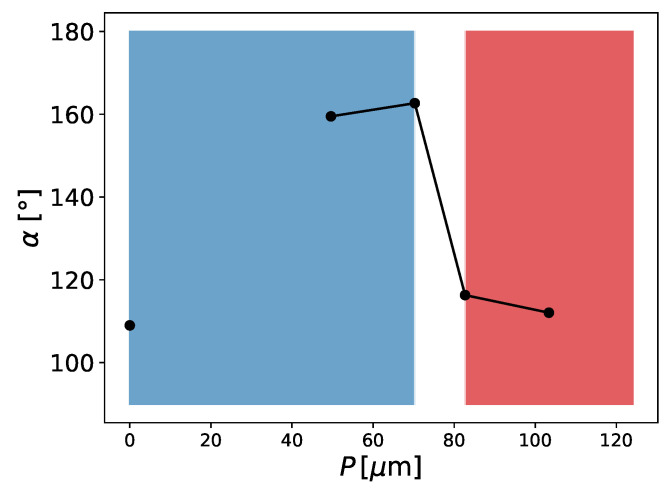
Contact angle α in dependence of the pitch between pillars (*P*). For the blue area, the droplet is in the Cassie–Baxter state and for the red area, the droplet is in the Wenzel state; 1lu≡4.135μm, droplet volume is 1μL, H=33μm, and $A_{top}=153.88\;\mu m^{2}$.

In this paper, we first analysed the dependence of the droplet state on the pillar structure (pillar distance) and the initial condition and compared the simulation results to the experiments performed by Jung and Bhushan [10] (Figure 6, Figure 7 and Figure 8). Similar to the 3D LB simulations performed by Xiong and Cheng [32], who also used a Shan–Chen-like model, we compared the apparent contact angle of water droplets on a rough micropillar structure to the theoretical predictions from the CB and Wenzel Equations (12) and (13).

We then performed a series of simulations with different starting conditions and different pillar distances *P*. The CB state is, in contrast to the Wenzel state, a meta-stable equilibrium state, where small perturbations can lead to a switch from the CB to the Wenzel state [18]. This is something we observed in our simulations, where different initial states led to different equilibrium states for certain geometries. Two initial states were tested. The first was a “cubic droplet” and the second was a spherical droplet, both above the rough surface. Similar to the work of Peng et al. [22], we used Equation (14) to initialise the spherical droplet. For P=12lu, both initial states led to a CB state. For P=17lu, only the spherical droplet led to a CB state and the cubical droplet led to a Wenzel state.

The simulation results in Figure 8 were initialised with a spherical droplet. At about P=70μm, we observed a transition from the CB to the Wenzel state. The geometries shown in Figure 8 are similar to the experimental results of Jung and Bhushan [10]. The droplet volume in the experiment was about 5μL and was larger than the present droplets (1μL) but still of the same magnitude. The reasons for this discrepancy are the computational limitations and long convergence times. The two contact angles in the Cassie–Baxter state are very similar to the observed values in the experiment, e.g., for P=70μm, we observed $162.68^{\circ }$ and Jung and Bhushan [10] observed about $163^{\circ }$. The transition from the CB state to the Wenzel state occurred between P=70μm and P=82μm (see Figure 8), whereas Jung and Bhushan [10] observed the transition at about P=130μm. This difference might be explained by the smaller size of the droplets and the difference in the surface tension, which in our case was 0.00022 N/m (La=75), being two orders of magnitude smaller than the surface tension of water. On this basis, one can conclude that the surface tension does not have a big effect on the apparent contact angle in the CB or Wenzel states. This is in agreement with Equations (12) and (13), which are independent of the surface tension. Furthermore, one can conclude that besides the initial condition and pillar distance, the surface tension also seems to have a significant impact on the state of the droplet (either CB or Wenzel).

(14) $$\rho \left(x,y,z\right)=\frac{\rho_{l}+\rho_{g}}{2}+\frac{\rho_{g}-\rho_{l}}{2}\cdot \tanh\left(\frac{2\left[\sqrt{{\left(x-x_{0}\right)}^{2}+\left(y-y_{0}\right)+{\left(z-z_{0}\right)}^{2}}-r\right]}{5}\right)$$

**Figure 9:**
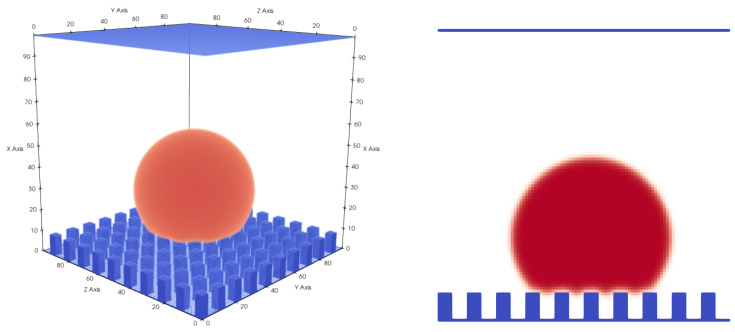
Droplet on a rough hydrophobic surface in the Cassie–Baxter state, with a total domain size of 100×100×100 voxels. Gaseous phase is not shown for better visibility. The roughness of the surface has an impact on the apparent contact angle, which in this case is $130.82^{\circ }$ ($G_{ads}=-157.16$, G=−120.0, P=10, H=9, $A_{top}=5\times 5\;{\mathrm{lu}}^{2}$).

Table 3 shows a comparison of our results for the apparent contact angle ($\alpha_{app}$) obtained with LB simulations to the theoretical values ($\alpha_{app}^{theor}$) calculated with the CB or Wenzel equations (Equations (12) and (13), respectively). Examples for the simulation results can be seen in Figure 6, Figure 7 and Figure 9.

**Figure 10:**
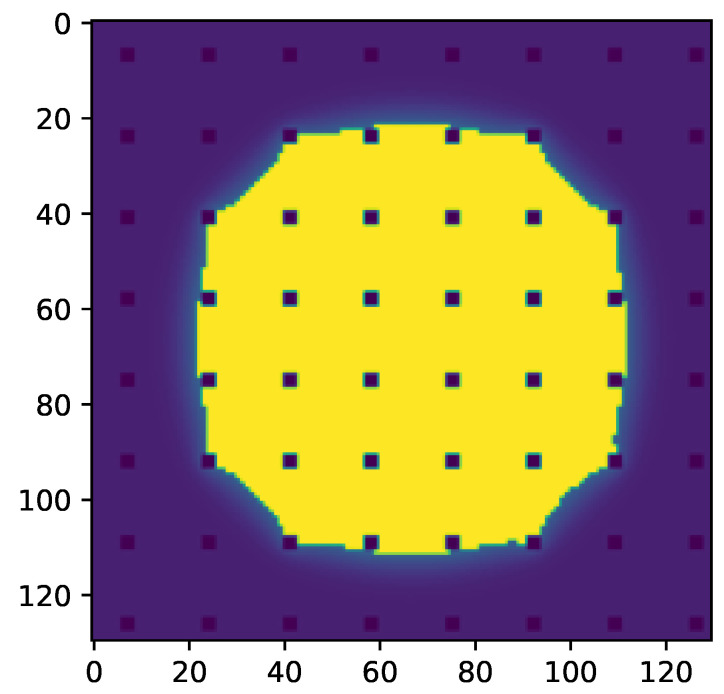
Slice through the droplet parallel to the surface right above the micropillars of the results seen in Figure 6. Liquid phase is shown in yellow and pillars are shown in dark blue. The yellow surface area can be used to calculate $f_{SL}^{*}$.

To calculate the theoretical apparent contact angle in the CB state using the CB equation, one needs to determine $f_{SL}$, the fraction of the bottom surface of the liquid droplet in contact with the solid. Based on the micropillar structure, one can estimate $f_{SL}=\frac{A_{top}}{P^{2}}$. Alternatively, one can use the yellow surface in Figure 10 to determine the fraction of the bottom surface of the liquid droplet $f_{SL}^{*}$ in contact with the solid more precisely, leading to slightly higher values (see Table 3). Using the simulation results to calculate $f_{SL}^{*}$, the CB equation predicted the apparent contact angles $\alpha_{app}^{theor,*}$ which were closer to our simulation results (see Table 3).

In general, it was observed that the predictions using the CB equation were closer to our simulation results for larger ratios of droplet sizes over *P*. This indicates that the difference between the CB equation and our simulations became larger as fewer pillars were in contact with the droplet. For higher hydrophobicities, the predictions of the CB equation were closer to our simulation results. This could indicate that the apparent contact angle also depends on other parameters such as the hydrophobicity (nanostructure) of the material and the droplet size. The simulation with a size of 121×175×175, as seen in Table 3, was equivalent to the geometry used by Xiong and Cheng [32]. They observed a contact angle of $135^{\circ }$ for the rough surface, which was about $3^{\circ }$ higher than our simulation results but also about $3^{\circ }$ below the predictions from the Cassie–Baxter equation. It should be noted that, in general, our contact angle measurements were highly sensitive to the location of the reference plane. Changing the location of the reference plane by one voxel could already lead to a $3^{\circ }$ difference in the contact angle.

### 3.4. Liquid Entry Pressure

#### 3.4.1. Cylindrical Pore

The liquid entry pressure (LEP) is the pressure difference between the hot water channel and the air gap, which is needed to make the water enter the pores of the hydrophobic membrane. For a perfect cylindrical pore with a radius ($r_{c}$), the LEP can be calculated using the following formula [33]:

(15) $$\mathrm{LEP}=\frac{-2\gamma \cos\beta_{0}}{r_{c}}$$

$\beta_{0}$ is the contact angle of a water droplet on the flat surface of the membrane material. With increasing pressure difference, the contact angle starts increasing until it reaches the critical value of $\beta_{0}$. After this point, the membrane material can no longer prevent the intrusion of water. The formula for the LEP can be easily derived from the Young–Laplace equation ($\Delta p=\frac{2\gamma }{r}$), where *r* is the radius of the spherical meniscus, by using $-\cos\beta =\frac{r_{c}}{r}$. This formula is only correct if the pressure in the meniscus is constant.

**Figure 11:**
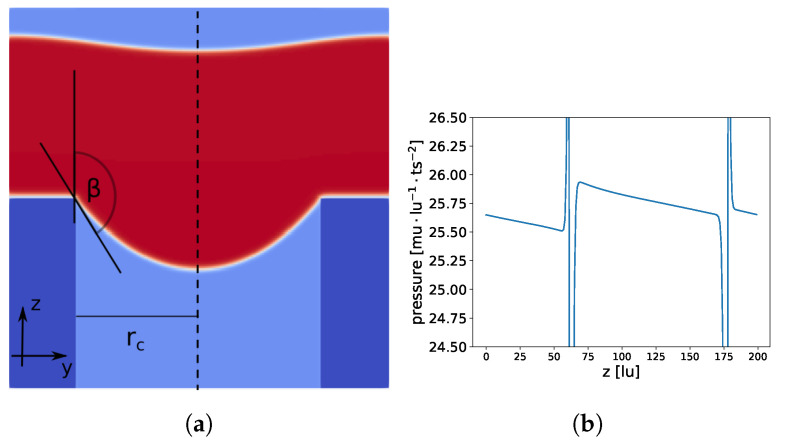
Constant pressure gradient is applied along the z-axis (from bottom to top) in the full domain. Total domain size is 200×200×200 lu${}^{3}$ and the pore radius is 64 lu. In (**a**), a slice at x=100 is shown; membrane material is dark blue, liquid is red, and gas is light blue. In (**b**), the pressure profile at the center of the cylindrical pore (dashed line in (**a**) at x=100, y=100) is shown.

To force the water to enter the membrane, we applied a constant pressure gradient along the direction that the water flow was moving (z-axis) over the full domain. This led to a non-constant pressure field in the meniscus and ultimately to a non-spherical meniscus shape (see Figure 11). The pressure decreased along the z-axis (excluding pressure jumps at the liquid–gas interfaces), which stretched the meniscus in the z-direction, leading to a “ellipsoidal like” meniscus. Lubarda and Talke [34], who analysed the equilibrium droplet shape for a similar problem (droplet on a flat surface in a gravitational field), found ellipsoidal droplets under certain conditions, supporting our observations of an ellipsoidal meniscus shape.

With $\frac{\rho \left(x,y,z\right)-\rho_{c}}{\rho_{c}}<0.01$, we extracted the points in the vicinity of the liquid–gas interface and fitted these points to two geometrical models, a spherical $\frac{{\left(x-x_{c}\right)}^{2}+{\left(y-y_{c}\right)}^{2}+{\left(z-z_{c}\right)}^{2}}{r^{2}}=1$ and an ellipsoid $\frac{{\left(x-x_{c}\right)}^{2}}{a^{2}}+\frac{{\left(y-y_{c}\right)}^{2}}{a^{2}}+\frac{{\left(z-z_{c}\right)}^{2}}{b^{2}}=1$. Using the ellipsoid model led to a more than one order of magnitude lower root mean square (RMS) deviation from the data points: ${\mathrm{RMS}}_{ellipsoid}=2.8\cdot 10^{-4}$ ($3.2\cdot 10^{-4}$) compared to ${\mathrm{RMS}}_{sphere}=7.2\cdot 10^{-3}$ ($7.3\cdot 10^{-3}$). The values in parentheses show the RMS for a set of 20% data points (randomly chosen), which were not used for the previous calculation, to check if overfitting occurred. In addition, the lengths of the axes a=106.89 and b=188.75 deviated significantly from r=69.12. This indicates that we indeed had an ellipsoidal-like meniscus shape rather than a spherical one.

**Figure 12:**
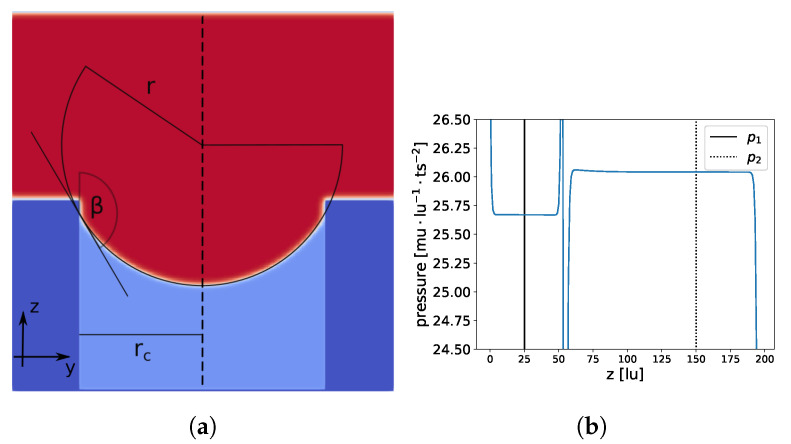
Constant pressure in liquid and gas phases. Total domain size is 200×200×200 lu${}^{3}$ and the pore radius is 64 lu. In (**a**), a slice at x=100 is shown; membrane material is dark blue, liquid is red, and gas is light blue. In (**b**), the pressure profile at the center of the cylindrical pore (dashed line in (**a**) at x=100, y=100) is shown.

A second simulation was also executed, setting a nearly constant pressure (density) for the liquid and gas phases (see Figure 12). To determine the LEP of a cylindrical pore, we chose the following setup: we removed the PBC in the z-direction (the direction of the water movement) and closed our domain at z=0 and z=199, initialized the fluid above the pore with a liquid density higher than the equilibrium liquid density, and chose $\rho_{l}=559.4$ for the upper half of the domain z>99. The pore was initialized with the equilibrium density for gas $\rho_{g}=85.7$ (lower half of the domain z<100). Initially, the liquid had a higher pressure, which led to an expansion of the liquid into the cylindrical pore. The pressure in the liquid started to decrease and a meniscus began to form, increasing the contact angle. If the initial pressure was sufficiently high, the critical angle $\beta_{0}$ was reached and the liquid started to enter the cylinder. The liquid continued entering the pore until the pressure difference between the liquid and gas was equal to the LEP.

The RMS for the spherical model ${\mathrm{RMS}}_{sphere}=2.1\cdot 10^{-3}\left(2.2\cdot 10^{-3}\right)$ and the ellipsoidal model ${\mathrm{RMS}}_{ellipsoid}=1.4\cdot 10^{-3}\left(1.5\cdot 10^{-3}\right)$ were of the same order of magnitude, and a=77.89 and b=82.91 deviated only slightly from r=75.08, indicating that a spherical model can be applied. The values in parentheses show the RMS for a set of 20% data points (randomly chosen), which were not used for the previous calculation, to check if overfitting occurred. In addition, the contact angle $\beta_{0}=148.48$ was similar to the contact angle of a droplet on a flat surface ($148.25^{\circ }$). The contact angle $\beta_{0}$ was calculated with $\cos\left(\beta_{0}\right)=-\frac{r_{c}}{r}$. Finally, we compared the LEP from our LB simulation $\Delta p=0.3716\;\mathrm{mu}\cdot {\mathrm{lu}}^{-1}\cdot {\mathrm{ts}}^{-2}$ to the value from Equation (15) $\Delta p_{pred}=0.3740\;\mathrm{mu}\cdot {\mathrm{lu}}^{-1}\cdot {\mathrm{ts}}^{-2}$. The small deviation can be explained by the discrepancies between the theoretical values of the surface tension and the contact angle, as shown in the Young–Laplace and wettability benchmarks. Overall, we found very good agreement between the LEP from the LB simulations and the predictions from Equation (15).

**Figure 13:**
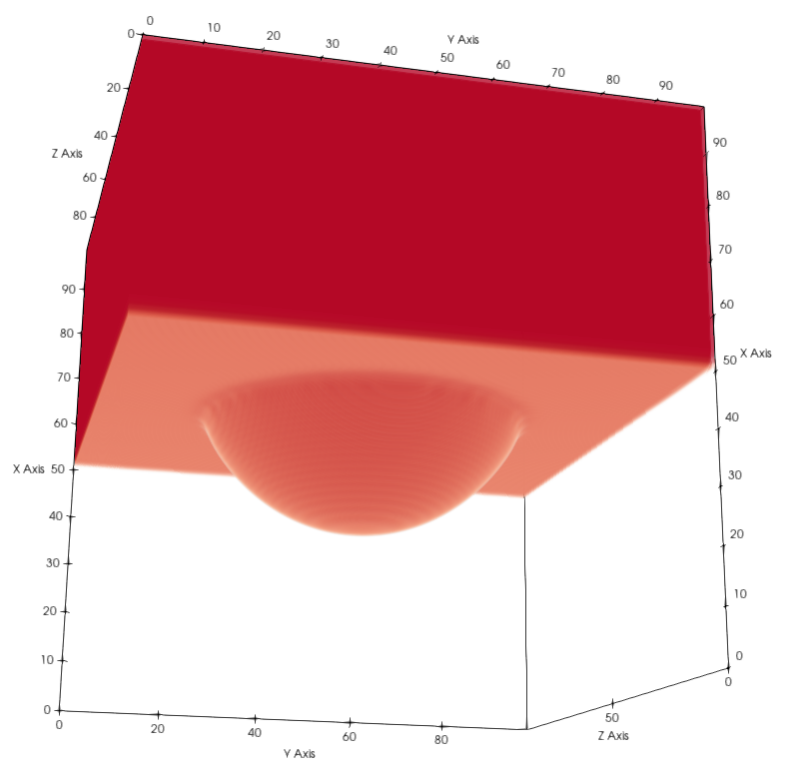
Liquid (red) entering a cylindrical pore. Gaseous phase and solid voxels are not shown for better visibility. We chose for this test $G_{ads}=-75.0$ and G=−180.0.

To validate the density (pressure) BC mentioned in the Methods section, we fixed the density at x=0 and $x=x_{max}$. According to the equation of state (see Equation (7)), this will also prescribe the pressure and the phase at x=0 and $x=x_{max}$. We used these BC to apply a pressure difference to one cylindrical pore. The BC in the *y*- and *z*-directions were periodic (see Figure 13).

The pressure difference can be calculated from the radius of the spherical meniscus and the surface tension as $\Delta p_{cal}=\frac{2\gamma }{r}=4.2468\;\mathrm{mu}\cdot {\mathrm{lu}}^{-1}\cdot {\mathrm{ts}}^{-2}$ and the prescribed pressure difference through the density BC as $\Delta p=p\left(\rho \left(x=0\right)\right)-p\left(\rho \left(x=x_{max}\right)\right)=4.2302\;\mathrm{mu}\cdot {\mathrm{lu}}^{-1}\cdot {\mathrm{ts}}^{-2}$. The radius *r* can be calculated by a fit similar to the geometrical models described above. The small difference between the pressure differences can be explained by the uncertainty of the radius.

#### 3.4.2. Realistic Distillation Membrane

**Figure 14:**
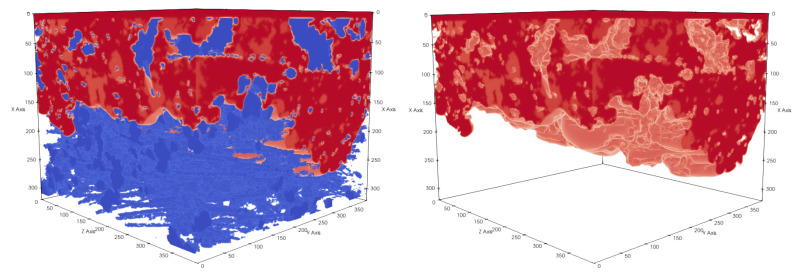
Subsection of sample 3 with membrane dimensions $11.7\times 15.6\times 15.6\;\mu m^{3}$. The pressure difference between the top and bottom sides of a membrane is Δp=1.320bar. Membrane material is shown in blue and liquid in red. Liquid did not completely cross the membrane geometry.

**Figure 15:**
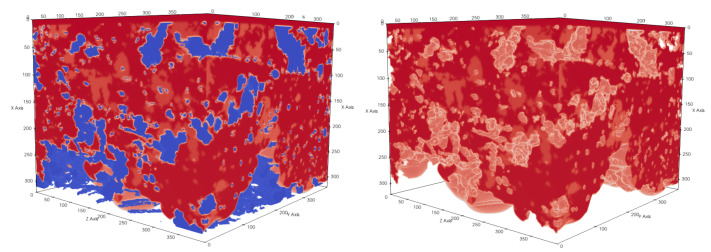
Subsection of sample 3 with membrane dimensions $11.7\times 15.6\times 15.6\;\mu m^{3}$. The pressure difference between the top and bottom sides of a membrane is Δp=1.912bar. Membrane material is shown in blue and liquid in red. Liquid fully crossed the membrane geometry.

**Figure 16:**
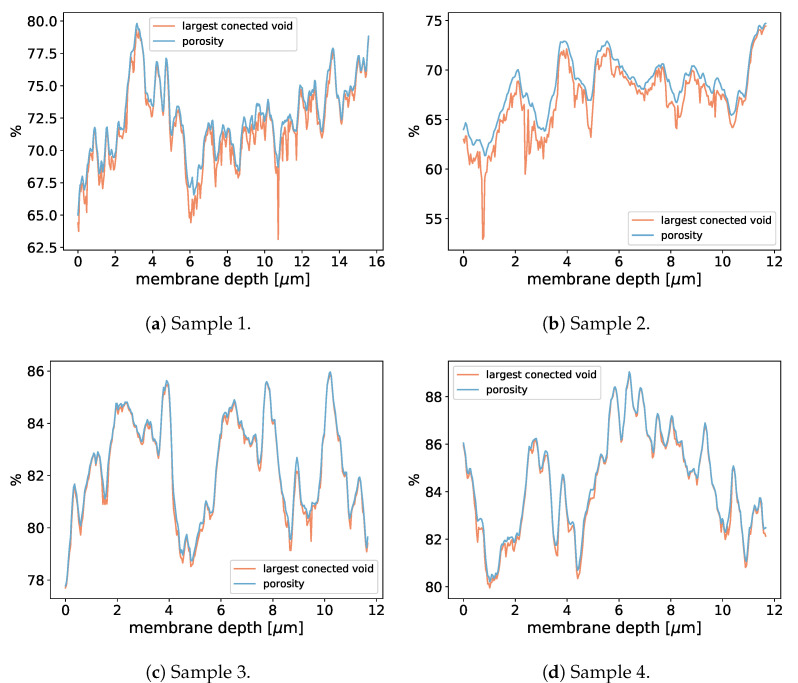
Porosity in dependence of the membrane depth (*x*-direction) for the the membrane subsamples used in Table 4.

To investigate the LEP of realistic membranes, we applied the aforementioned pressure boundary conditions with multiple pressure differences, as shown in Table 4, Figure 14 and Figure 15, to realistic distillation membranes. The space above the membrane material was initially filled with liquid and the rest of the domain with gas. Because of the pressure difference between x=0 and $x=x_{max}$, the liquid started to penetrate the membrane structure. Depending on the magnitude of the pressure difference, we observed different liquid entry depths (LED) into the membrane structures, as shown in Table 4.Therefore, we were able to determine the breakthrough pressure of the membrane subsamples. Since the LED depends on the porosity we include the porosity profils of the invesigated subsamples in Figure 16.

For the simulations with the membrane geometry, we chose $G_{ads}=-275.0$, which corresponds to a contact angle of about $105^{\circ }$, which is the contact angle reported experimentally for a water droplet on a flat PTFE surface [35,36]. We reported the pressure differences and the LEP for different membrane subsamples in Table 4. The results are in good agreement with the literature (measured experimentally and the values from the manufacturer). According to Racz et al. [33], the LEP for these membranes lies between 1 and 4 bar (see Table 1). The LEP for samples 1 and 4 was 2.8bar and the LEP for sample 2 was 3.68bar [33]. Since we used a thin membrane sample, we expected the calculated LEP to be below the value in the literature.

A necessary remark is that our model does not represent the correct density ratios between the liquid and gaseous phases (See Table 2). Since we were not interested in the dynamics of the entering process but rather whether a membrane breakthrough occurred, the mismatch of the liquid–gas density ratio should not have significantly affected the results. Due to the high hydrophobicity, the water entered the membrane relatively slowly. The liquid-entering depth was mainly governed by the contact angle (through the hydrophobicity) of the material and the surface tension of the liquid. To verify this, we calculated the liquid-entering velocity and the capillary number for the entering process ($v_{enter}=1.5\;\mathrm{cm}/s$ and Ca=0.00022). The very small value of the capillary number shows that the surface tension forces are far more important than the viscous forces, similar to the Bond number and the gravity forces mentioned previously.

**Figure 17:**
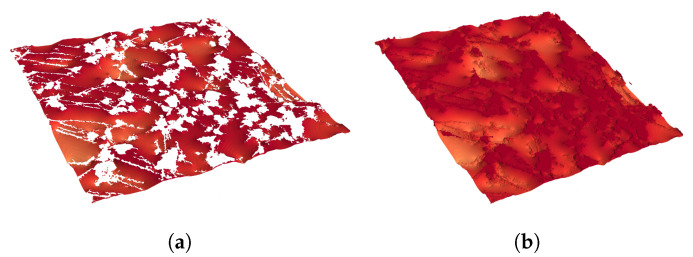
Example of the liquid–gas contact area in (**a**) and the liquid surface area in (**b**).

An important property for membrane distillation is the surface area of the liquid–gas interface since it will have an impact on the evaporation flux. With the current model, we were able to determine the liquid–gas surface areas for our membrane samples (See Figure 17). We calculated the areas in Table 5 and Table 6 with the marching cubes algorithm [37] implemented in the Python skimage library [38]. Because recent work has shown that micropillars on membranes increase hydrophobicity and reduce scaling effects [9], we also added pillars on top of the membrane structure to investigate the influence on the liquid–membrane contact interface. The distance between the pillars was P=3.9μm, the pillar radius was 0.39μm, and the pillar height was H=1.95μm. The pillars only led to a small decrease in the liquid–gas contact surface area (see Table 5), which can be explained due to the fact that they blocked the pore space. Apart from that, the impact of the pillars seemed to be negligible for the liquid–gas surface area.

Comparing the liquid–gas area ($A_{l,g}/A_{yz}$) in Table 5 to the porosity in Figure 16, one can conclude that they correlate and that the porosity is the limiting factor for the liquid–gas interface.

### 3.5. Liquid–Vapour Interface within a Realistic Distillation Membrane

In MD distillation, the air in the membrane and the air gap are expected to be close to saturation, which makes condensation within the membrane material possible; therefore, we investigated the water droplet distribution within the membrane structure. Similar to Pot et al. [39], we initialized the membrane geometry with a homogeneous density between the equilibrium liquid and vapour density. As the simulation started, the two phases started to separate and the location of air–water interfaces was determined. Pot et al. [39] found a remarkably accurate prediction of the air–water interfaces within a porous medium by comparing the LB model to the experimental results. Depending on the initial mean density, different saturation states were realised.

The saturation for our simulations was $S=\frac{\rho_{init}-\rho_{l}}{\rho_{g}-\rho_{l}}\approx 10\%$. For the following simulations, we again chose $G_{ads}=-275.0$ and G=−180.0. The density field was initialized with a mean density of $\rho_{init}=150\;\mathrm{mu}\;{\mathrm{lu}}^{-3}$ with a random perturbation of up to 1%, leading to static phase separation. This allowed us to determine the water droplet agglomeration spots in the membrane structure. In Table 6, we reported some characteristics of the droplet distribution. The last column in Table 6 shows the ratio of the surface area of all liquid droplets to a single sphere with the same volume. This value correlates with the number of droplets and the droplet shape, showing that more droplets lead to a higher value.

**Figure 18:**
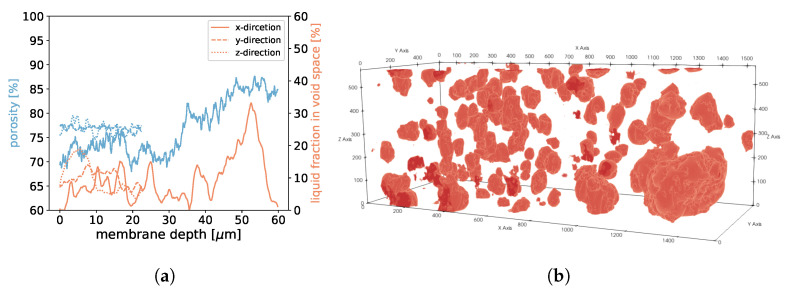
In (**a**), the porosities along the *x*-, *y*-, and *z*-axes are shown for sample 1. Moreover, we display the fraction of void space that is occupied by liquid at a given membrane depth. In (**b**), liquid droplets are shown in red for sample 1. Gaseous phase and membrane material is not shown for better visibility. It should be noted that the convergence criterion in Equation (11) only reached $4.6\cdot 10^{-5}$.

**Figure 19:**
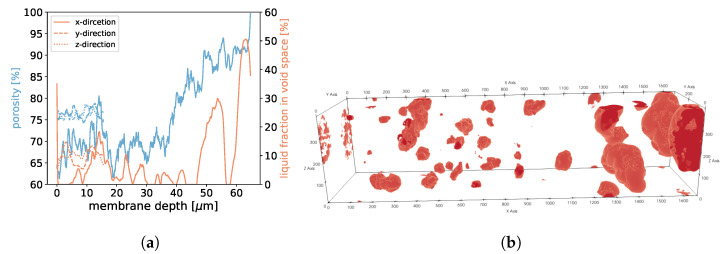
In (**a**), the porosities along the *x*-, *y*-, and *z*-axes are shown for sample 2. Moreover, we display the fraction of void space that is occupied by liquid at a given membrane depth. In (**b**), liquid droplets are shown in red for sample 2. Gaseous phase and membrane material is not shown for better visibility. It should be noted that the convergence criterion in Equation (11) only reached $1.7\cdot 10^{-5}$.

**Figure 20:**
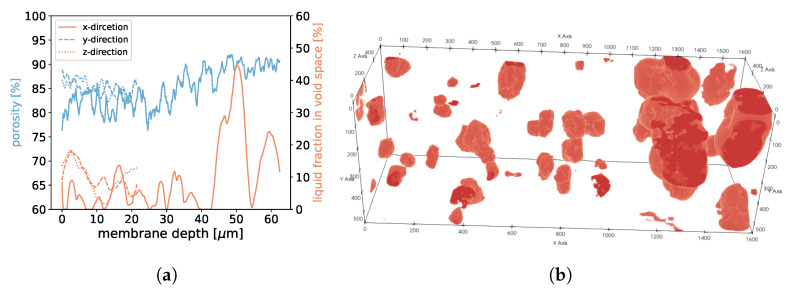
In (**a**), the porosities along the *x*-, *y*-, and *z*-axes are shown for sample 3. Moreover, we display the fraction of void space that is occupied by liquid at a given membrane depth. In (**b**), liquid droplets are shown in red for sample 3. Gaseous phase and membrane material is not shown for better visibility.

**Figure 21:**
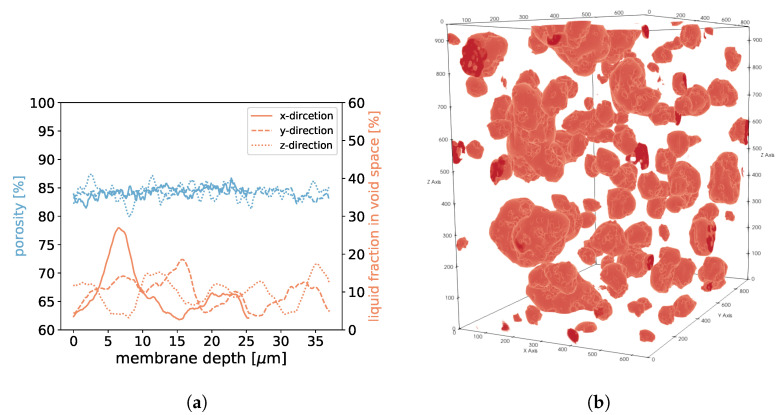
In (**a**), the porosities along the *x*-, *y*-, and *z*-axes are shown for sample 4. Moreover, we display the fraction of void space that is occupied by liquid at a given membrane depth. In (**b**), liquid droplets are shown in red for sample 4. Gaseous phase and membrane material is not shown for better visibility. It should be noted that the convergence criterion in Equation (11) only reached $3.8\cdot 10^{-5}$.

For samples 1, 2, and 3, we found that in areas of higher porosity, the agglomerated droplets were bigger compared to areas with low porosity (See Figure 18, Figure 19 and Figure 20). In addition, more liquid was agglomerated in the areas of high porosity. If water droplets were formed during MD in the center of the membrane structure, it was likely that the performance decreased because of the blockage of the pores. To avoid such a scenario, a positive gradient in the porosity of the membrane material, as was the case for samples 1, 2, and 3, could be beneficial since droplet formation was more likely at the end of the membrane and not in the center. Sample 4 had the most homogeneous distribution of droplets, which was in line with the homogeneous porosity of this sample (See Figure 21).

The presented multi-phase model predicted where water droplets agglomerated if condensation took place. When droplets are formed, two processes are competing. One process is the minimisation of the surface area due to the surface tension and the other process is linked to the hydrophobicity of the membrane material. Since we are dealing with hydrophobic membrane material, the liquid has a higher chance of preferentially concentrating in high-porosity areas because more space is available and less liquid is in contact with the membrane material. Condensation can happen at nucleation sites in the steam or on the membrane surface. The hydrophobic membrane structure and the gradient in the porosity could transport small droplets towards areas of high porosity during the formation process.

## 4. Conclusions

We performed 3D multi-phase LB simulations to gain an understanding of the phase distribution within the microporous structure of a realistic distillation membrane. We validated the model, showing good agreement with various benchmarks. Besides the typical benchmarks such as the Laplace law and the contact angle benchmark on a flat surface, we also validated the applicability to more complex membrane geometries. In particular, we validated the apparent contact angle of droplets on a rough micropillar structure against theoretical predictions and compared the LEP of our model to the values of the manufacturers and found good agreement for these benchmarks. Finally, we were able to determine the realistic contact surfaces between typical distillation membranes and water at the pore level. Additionally, we determined the air–water interface within a partially saturated membrane, finding that the droplet size and distribution correlated with the porosity of the membrane.

In future works, the surface areas reported in Table 5 and Figure 17 can be used as boundary conditions for a multi-component LB model including the evaporation and diffusion of water vapour through the porous membrane material. More investigation is needed on the influence of micropillars and flow on the liquid–gas interface. For the pillars and flow tested in this work, we found that they did not significantly influence the shape of the liquid–gas interface (see Table 5). Different micropillar structures and coatings with different hydrophobicities can be added easily to the membrane structures in future works, which can then be used to optimize the membrane structure to increase the evaporation flux.

Understanding the underlying interactions at the pore level within the membranes, as well as the interaction of membrane surface characteristics and mass transport mechanisms, is key to optimizing desalination units that work on the principle of membrane distillation. Within the framework of the lattice Boltzmann method, other physical mechanisms may be added in the future such as the precipitation and transport of contaminants within the pore structure.

## Figures and Tables

**Table 1:** 3D membrane geometries obtained by Cramer et al. [12].

Sample	Membrane	Manufacturer	Pore Diameter	Sample Dimensions (x×y×z)	
			[μm]	[$\mu m^{3}$]	[Voxels]
1	FGLP14250	Merck Millipore	0.2	59.85 ×22.42×22.42	1535×575×575
2	Gore ${}^{a}$	Gore	0.22	57.70×15.60×15.60	1480×400×400
3	FHLP14250	Merck Millipore	0.45	62.42×21.44×21.44	1601×550×550
4	FGLP14250	Merck Millipore	0.2	25.34×37.04×37.04	650×950×950

**Table 2:** Summary of membrane and fluid properties.

	LB Units	SI Units	Remark
$\rho_{l}$	1028	998.861 $\frac{\mathrm{kg}}{m^{3}}$	pure water, 1 bar, 16.5 ${}^{\circ }$C
$\rho_{g}$	53	1.204 $\frac{\mathrm{kg}}{m^{3}}$	1 bar, 16.5 ${}^{\circ }$C
Δρ	975	997.657 $\frac{\mathrm{kg}}{m^{3}}$	
$\rho_{l}/\rho_{g}$	19.4	829.619	
$\mu_{l}$	171.33	$1.094\cdot 10^{-3}$ Pa·s	1 bar, 16.5 ${}^{\circ }$C
$\mu_{g}$	8.83	$1.81\cdot 10^{-5}$ Pa·s	1 bar, 16.5 ${}^{\circ }$C
$\mu_{l}/\mu_{g}$	19	60	
Δx	1	$0.039\cdot 10^{-6}$ m	
Δt	1	$0.23\cdot 10^{-9}$ s	
*c*	1	168.5 m/s	
La	24,424	24,424	1 bar, 16.5 ${}^{\circ }$C
γ	68	0.073 N/m	pure water, 1 bar, 16.5 ${}^{\circ }$C
Bo	$1\cdot 10^{-4}$	$1\cdot 10^{-4}$	

**Table 3:** The apparent contact angle $\alpha_{app}$ on a rough surface is compared to the predictions using the Cassie–Baxter (CB) and Wenzel equations. Values in parentheses show the deviations from the LB simulations as percentages.

State	Size [voxel${}^{3}$]	$V_{\mathrm{drop}}$ [voxel${}^{3}$]	P	H	α [${}^{\circ }$] Flat LBM	$\alpha_{\mathrm{app}}$ [${}^{\circ }$] Rough LBM	$\alpha_{\mathrm{app}}^{\mathrm{theor},*}$ [${}^{\circ }$]	$f_{\mathrm{SL}}^{*}$	$\alpha_{\mathrm{app}}^{\mathrm{theor}}$ [${}^{\circ }$]	$f_{\mathrm{SL}}$	$R_{f}$
CB	100 × 100 × 100	$9\cdot 10^{4}$	10	10	126.75	145.99	152.40	0.2834	154.10	0.25	-
							(4.4%)		(5.6%)		
CB	100 × 100 × 100	$9\cdot 10^{4}$	10	10	119.76	143.24	148.99	0.2838	150.94	0.25	-
							(4.0%)		(5.4%)		
CB	100 × 100 × 100	$9\cdot 10^{4}$	10	10	110.6	130.82	145.31	0.2675	146.93	0.25	-
							(11%)		(12%)		
CB	121 × 175 × 175	$5\cdot 10^{5}$	20	15	119.76	131.57	-	-	138.15	0.51	-
									(5.0%)		
CB	320 × 380 × 380	$8\cdot 10^{6}$	20	15	109	151.20	158.73	0.101	159.65	0.0925	-
							(5.0%)		(5.6%)		
CB	342 × 384 × 384	$14.7\cdot 10^{6}$	12	8	109	159.50	162.88	0.0657	163.31	0.0625	-
							(2.0%)		(2.3%)		
CB	357 × 374 × 374	$14.1\cdot 10^{6}$	17	8	109	162.68	167.465	0.0353	168.24	0.0311	-
							(3.0%)		(3.4%)		
Wenzel	340 × 360 × 360	$14.1\cdot 10^{6}$	20	8	109	116.90	-	-	113.81	-	1.24
									(−2.6%)		
Wenzel	357 × 374 × 374	$14.1\cdot 10^{6}$	25	8	109	111.58	-	-	112.06	-	1.1536
									(0.43%)		

**Table 4:** Liquid entry depth in the x-direction and pressure differences for multiple membrane subsamples obtained from the LB simulations are compared to the liquid entry pressures from the experiments. Predictions are in close agreement with the experimental values.

Sample	Membrane Dimensions (x×y×z)		Δp	Liquid Entry Depth in x	LEP Experiments [33]
	[$\mu m^{3}$]	[voxels]	[bar]	[μm]	[bar]
1	15.6×11.7×11.7	400×300×300	3.112	>15.6 (breakthrough)	2.8
	15.6×11.7×11.7	400×300×300	2.510	>15.6 (breakthrough)	(from manufacturer)
	15.6×11.7×11.7	400×300×300	1.912	2.262	
	15.6×11.7×11.7	400×300×300	0.732	0.897	
	3.9×19.5×19.5	100×500×500	0.182	0.975	
2	15.6×11.7×11.7	400×300×300	2.510	>15.6 (breakthrough)	3.68±0.01
	15.6×11.7×11.7	400×300×300	1.912	3.315	
	11.7×15.6×15.6	300×400×400	2.21	10.608	
	11.7×15.6×15.6	300×400×400	1.912	6.474	
	11.7×15.6×15.6	300×400×400	1.320	1.716	
	3.9×15.6×15.6	100×400×400	0.182	0.585	
3	11.7×15.6×15.6	300×400×400	1.912	>11.7 (breakthrough)	
	11.7×15.6×15.6	300×400×400	1.320	3.315	
	3.9×19.5×19.5	100×500×500	0.182	0.897	
4	11.7×15.6×15.6	300×400×400	1.912	>11.7 (breakthrough)	2.8
	11.7×15.6×15.6	300×400×400	1.320	>11.7 (breakthrough)	(from manufacturer)
	11.7×15.6×15.6	300×400×400	1.025	9.243	
	3.9×19.5×19.5	100×500×500	0.182	0.975	

**Table 5:** Liquid–gas contact area. Membrane subsample size is $3.9\times 19.5\times 19.5\;\mu m^{3}$ for sample 1, 3, and 4 and $3.9\times 15.6\times 15.6\;\mu m^{3}$ for sample 2. Δp=0.182bar. $A_{l,g}$ is the liquid–gas contact surface area, $A_{l}$ is the total surface area of the liquid, and $A_{yz}$ is the area of the membrane cross-section. The velocity *v* is introduced parallel to the membrane plane and at a distance of 5.85μm from the membrane plane.

Sample	*v* [cm/s]	Pillars	$A_{l,g}/A_{l}$ [%]	$A_{l,g}/A_{\mathrm{yz}}$ [%]	$A_{l}/A_{\mathrm{yz}}$ [%]
1	0.0	no	51.96	64.48	124.10
2	0.0	no	51.3	63.99	124.74
3	0.0	no	62.4	76.96	123.33
3	1.7	no	62.4	76.96	123.33
3	0.0	yes	44.24	71.73	162.14
3	1.7	yes	44.25	71.74	162.12
4	0.0	no	64.08	81.88	127.78

**Table 6:** Surface area between liquid and gaseous phases ($A_{l,g}$) compared to the surface area of a sphere with the same volume ($A_{sphere}$) and the total surface area of all droplets, which includes the surface area of the droplets that are in contact with the membrane material ($A_{l,gs}$).

Sample	Domain Dimensions	$V_{\mathrm{liquid}}$ [%]	$V_{\mathrm{gas}}$ [%]	$V_{\mathrm{solid}}$ [%]	$A_{l,g}/A_{l,\mathrm{gs}}$ [%]	$A_{l,g}/A_{\mathrm{sphere}}$ [%]	$A_{l,\mathrm{gs}}/A_{\mathrm{sphere}}$ [%]
1	1535 × 575 × 575	7.61	69.25	23.14	48.72	328.84	674.96
2	1660 × 400 × 400	7.57	68.8	23.63	57.66	222.17	385.31
3	1601 × 550 × 550	8.42	76.62	14.96	60.53	239.15	395.09
4	650 × 950 × 950	8.33	75.9	15.77	53.29	350.47	657.66

## Data Availability

The data presented in this study are available on request from the corresponding author.
